# The Effect of Hamstring Relaxation Program on Headache, Pressure Pain Threshold, and Range of Motion in Patients with Tension Headache: A Randomized Controlled Trial

**DOI:** 10.3390/ijerph181910137

**Published:** 2021-09-27

**Authors:** Soon-Hyun Kwon, Eun-Jung Chung, Jin Lee, Sang-Woo Kim, Byoung-Hee Lee

**Affiliations:** 1 Graduate School of Physical Therapy, Sahmyook University, Seoul 01795, Korea; yellow1496@hanmail.net (S.-H.K.); leejin87@hanmail.net (J.L.); 2 Department of Physical Therapy, Andong Science College, Andong 36616, Korea; qkskskzl@nate.com; 3 Virtual Rehabilitation Lab, Sahmyook University, Seoul 01795, Korea; adonis2023@naver.com; 4 Department of Physical Therapy, Sahmyook University, Seoul 01795, Korea

**Keywords:** headache, pain, hamstring, relaxation

## Abstract

The purpose of this study was to determine if the severity of headache is reduced by decreasing hamstring tension in patients with tension headache. Thirty patients participated in this study. The participants were randomly allocated to two groups: hamstring relaxation program (HR) group (*n* = 15) and control group (*n* = 15). The participants in the HR group participated in a HR program for 25 min per day, three times per week, for a period of 4 weeks, and the control group participated in an electrotherapy for 25 min per day, three times per week, for a period of 4 weeks. Both groups participated in a self-myofacial release for 5 min per day, three times per week, for a period of 4 weeks. Headache was evaluated using the headache impact test (HIT-6) and visual analog scale (VAS). The pain pressure threshold (PPT) was evaluated using a digital pressure algometer. The range of motion (ROM) was evaluated using a goniometer and two special tests: straight leg raise test (SLRT) and popliteal angle test (PAT). The two groups showed no significant differences in terms of age, sex, height, and weight. The VAS and HIT-6 scores (*p* < 0.05) and neck and hamstring PPT showed significant improvements (*p* < 0.05). Neck flexion ROM and SLRT and PAT scores showed significant improvements (*p* < 0.05) in both groups, and the HR group showed significantly more improvements than the control group. This study confirmed that the HR program has positive effects on tension headache and is a good intervention for alleviating headaches in patients with tension headache.

## 1. Introduction

The tension-type headache (TTH) suffers from a still unclear classification and equally unclear pathophysiological mechanisms. Several mechanisms have been proposed to be involved in its pathophysiology including vascular, peripheral (myofascial nociception) and central mechanisms (sensitization and inadequate endogenous pain control) [1,2]. Tension-type headache is described as a headache that evokes a sensation of the head being compressed or squeezed but without any underlying medical cause. It is mainly caused when the sympathetic nerve is provoked by abnormal autonomic nerves and constriction of blood vessels in the head and neck due to factors such as muscle tension, stress, fatigue, and lack of sleep [3]. A review of the prevalence of headaches worldwide and the burden of treatment for headaches reported that the average cost of direct and indirect treatments is 303 euros per person per year, besides the major influence on daily life [4]. The intensity and frequency of tension in the myofascial tissue of the muscles around the skull are found to be higher in patients with chronic tension headache than in healthy people [5]. In addition, when the muscles around the neck are tensed, the muscles in the limbs are also tensed [3]. 

The main cause of tension-type headache is pain caused by the myofascial trigger point of the neck musculature, which is MTrP of suboccipital muscles (rectus capitis posterior major, rectus capitis posterior minor, obliquus capitis superior, and obliquus capitis inferior) [6]. These abnormal muscle spasm of MTrP are closely related to forward head postures in which the head moves forward [7].

Interestingly, Gerwin [8] demonstrated that increased tension and shortening of the hamstring muscle can cause neck and shoulder pain. Hamstring and sub-occipital muscles are connected by a neural system and sub-occipital muscles pass through the dura mater [9]. This is called superficial back line (SBL), which connects the lower extremities, trunk, neck, and head protects the body’s entire posterior surface and provides an important function of up-righting the body [10]. The muscles and fascia contained in the SBL include plantar fascia, GCM, hamstring, erector spinae, epi-cranial fascia, and so on [11]. Therefore, it is probable that if the tone of the hamstring muscles is decreased (passively, with a fascial treatment or with active movements), the amplitude of hip flexion is increased, thereby increasing the straight leg raise (SLR) test score and the tone of the sub-occipital muscles is reduced [12]. 

Muscle chains connect the connective tissue fascia and muscles along specific lines of the body and help transport the epidural to the occipital muscle. Considering the effect of the occipital muscle restraint technique in treating hamstring tension, Aparicio et al. [13] hypothesized that the hamstring affects the postural control role of the occipital muscles. The occipital muscles are involved in postural control, and the hamstrings can influence the alignment of the body, and the importance of treating the upper cervical spine by occipital muscle suppression well known, but its relationship with other structures has not been examined [13]. In addition, a study on the effect of stretching the hamstrings on the tension in the muscle groups in the mandibular region in patients with jaw joint disorders also supports the theory that the relaxation of the hamstrings affects muscle tension and pain in the cervical region [14]. 

The hamstring is the largest muscle in the SBL, which creates the greatest tension in the body during tension, causing the connected pelvis to be posterior tilt so that it can be flat back posture or sway back posture [15]. The muscle is the main cause of being a slumped posture in a sitting position [16]. The flat back posture, sway back posture in the standing position, and the slumped posture in the sitting position is protected (or moved forward) by allowing the lower cervical to be flexed and the upper cervical to be extended [15,17]. Causing abnormal spasm of other neck musculature including suboccipital muscles, resulting in tension-type headache [7]. Therefore, securing flexibility of hamstring with physical therapy interventions for tension-type headache is a very important factor [12].

However, there is still insufficient research on the functional limitations of the occipital muscles and hamstrings in patients with tension headache. Therefore, in this study, investigated the effect of hamstring relaxation programs on the patient’s headache, suboccipital muscle range of motion (ROM) and pressure pain threshold (PPT), and hamstring muscle ROM and the PPT for tension headache. This supports the necessity of and provides an effective intervention method for headaches in patients with tension headache.

## 2. Materials and Methods

### 2.1. Subjects

In all, 30 generally healthy adults (15 men and 15 women) in their 20 s and 50 s who visited the H Rehabilitation Department in Seoul for a headache were selected. The inclusion criteria were as follows: diagnosed with tension headache, complaint of headache in the last 4 weeks, headache lasting for more than 30 min, 59 points or more on the headache effect test, and result of less than 80° on the straight leg raise test (SLRT). The exclusion criteria were history of spinal surgery, shoulder surgery, and traffic accidents and participation in other similar experiments. Additionally, subjects who have been diagnosed with psychiatric comorbidity or who have taken psychotropic drugs over the past thirty days were excluded. The purpose and details of the study were explained to all subjects who participated in the study, and consent was obtained after explaining that participation in the study could be withdrawn at any time during the study. This study was conducted with the approval of the Research Institutional Review Board of Sahmyook University (approval number: 2-7001793-AB-N-012018058HR), and it was registered (KCT0005811) on Clinical Research Information Service (CRIS) in Republic of Korea. The objective and the procedures to be performed in the study were fully understood by the subjects, and all subjects provided informed consent for inclusion in the study. Therefore, this study was based on the ethical principles of the Declaration of Helsinki.

### 2.2. Experimental Procedure

Subjects were selected for this study based on the inclusion or exclusion criteria. Before recruiting participants for this study, we performed a power analysis using G*Power version 3.1.9.7 (Heinrich-Heine-Universität, Düsseldorf, Germany), the overall effect size index for all the outcome measures and power of the study were 0.53, a probability of 0.05, and to minimize type II error (power of 80%). Because the estimated target sample size was 30, we recruited 30 participants for this experiment. Pretests were performed a week before the program started. 

**Figure 1:**
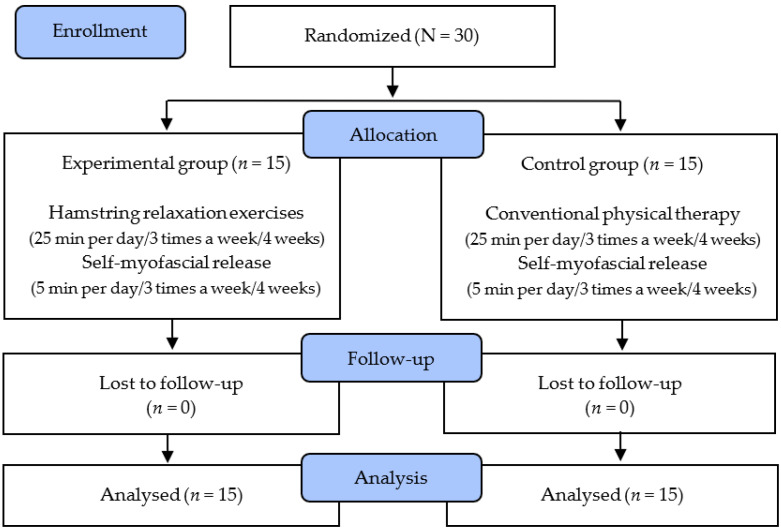
Flow diagram of total experimental procedure.

The study subjects selected 30 patients with tension headaches who initially indicated their willingness to participate, and randomly assigned them to the hamstring relaxation program (HR) group (*n* = 15) and control group (*n* = 15). Before the experiment, the subjects were asked to report their general characteristics such as age, height, and weight directly through the questionnaire. The degree of headache, PPT and range of motion (ROM) of the occipital muscle, and PPT and ROM of the hamstrings were measured. All subjects received treatment three times a week for 4 weeks, where the HR program was administered to the HR group and the control group was treated with electrotherapy. The subjects in the HR group used a foam roller for 5 min for self-myofascial release of the hamstrings; then, they performed HR exercises involving stretching of the proximal and distal parts of the hamstrings, considering the open and closed chains, for 25 min. The control group underwent interferometric current treatment and infrared heat treatment simultaneously for 25 min after self-myofascial release of the hamstrings using a foam roller for 5 min. After four weeks of the intervention, the degree of headache and the PPT and ROM of the occipital muscles and the hamstrings were measured again. The study protocol is depicted in the following chart (Figure 1).

### 2.3. Training Program

#### 2.3.1. Hamstring Relaxation Program

HR training was applied to stretch the hamstrings in the proximal and distal parts, considering the open and closed chains three times a week for 25 min for a total of 4 weeks. After the first 30 s of rest, the patient would bend the hip to stretch without flexing the knee in the supine position so that the proximal part of the hamstring was stretched in the open chain. 

Each set included 10 s of stretching and 5 s of rest for a total of 2 min and 30 s. After 30 s of rest, the patient would lie in the supine position and flex the leg to 90°, bend the knee to 90°, and fix the hip and knee so that the distal part of the hamstring was stretched in the open chain. The action was performed for 10 s of stretching and 5 s of rest for a total of 2 min and 30 s. After 30 s of rest, the patient placed the leg that was not to be stretched on the floor in a straight line, so that closed-chain stretching was achieved in the proximal part of the hamstring of the other leg, and the upper body was slowly bent forward while maintaining the knee joint in extended position. After 30 s of rest, the patient would flex the trunk to 45°, placing the leg to be stretched on the bench, with the knee joint naturally bent and the entire sole brought into contact with the bench, so that closed-chain stretching was achieved in the proximal part of the hamstring of the contralateral leg. After that, with one foot on the floor, the knee joint of the contralateral leg was extended. The action was performed for 10 s of stretching and 5 s of rest for a total of 2 min and 30 s. The sets were performed on both the left and right sides [18].

#### 2.3.2. Conventional Physical Therapy

Conventional physical therapy included electrotherapy. The patient was placed in a prone position to minimize tension in the hamstring, and transdermal neurostimulation treatment was applied to the hamstring. A frequency of 5–1000 Hz was used; the electric shock voltage was 220 V AC, the frequency was 60 Hz, and low-frequency-high-intensity stimulus (10–20 Hz) was set. The applied intensity was measured on the basis of the intensity of the visible contraction and absence of pain [19]. This therapy was administered for a total of 4 weeks, three times a week for 25 min. 

#### 2.3.3. Self-Myofascial Release

During the study, to prevent injury and damage to the patient, self-myofascial release of hamstrings was performed using the weight and gravity of the patient in a supine position with a foam roller. Fascia relaxation was performed by placing the hamstrings on a foam roller and moving the legs from side-to-side whilst lying down. It was applied to the proximal, middle, and distal parts of the hamstring, and each part was allowed to relax for 95 s and rest for 5 s. 

### 2.4. Outcome Measures

The headache impact test (HIT-6) and visual pain scale (VAS) were used to evaluate the intensity of the headache. The HIT-6 is a tool that subjectively evaluates the frequency of a patient’s headache. The lowest score is 36, and the highest score is 78 for six items. Usually, if the score is over 59, it means the patient’s daily life is severely affected by the headache; this test was developed to measure the effect of not only migraine but also other types of headaches, with a reliability of 0.85 [20]. 

The VAS is a tool that subjectively evaluates the intensity at which a patient feels pain and the degree of pain is expressed as 0–10 points, where 0 points = no pain and 10 points = unimaginable pain). The VAS is a simple and highly reproducible scale with a reliability of 0.99 [21]. 

A digital pressure pain gauge (PainTest™ FPX 25 Algometer; Wagner Instruments, Riverside, CT, USA, 2015) was used to determine the pain threshold of the inferior larynx and hamstring. In the prone position, the physician applied 1 kg/s of pressure directly to the suboccipital muscle, and the patient spoke up at the point where the pressure evoked a painful sensation, and the instantaneous value was recorded as the PPT. The experiment was conducted on both the left and right sides, and after a total of three measurements, the average value was calculated. The higher the average value, the lower the PPT [22]. The PPT of the hamstrings was also used to measure using a pressure algometer. In the prone position, the physician applied 1 kg/s of pressure directly and vertically to the hamstring, and the patient was asked to speak up at the point at which the pressure evoked a painful sensation, and the instantaneous value was recorded as the PPT. The experiment was conducted on both sides, and after a total of three measurements, the average value was calculated. A higher average value indicated a lower PPT, with a reliability of 0.89 [22]. All measurements were taken before and after all programs according to the therapeutic schedule.

The ROM of the suboccipital muscle was measured using an electronic goniometer to measure the flexion range of the neck in the supine position. The ROM hamstrings was measured using two special tests: the SLRT and the popliteal angle test [23]. In the SLRT, the subject was placed in a supine position, the patient’s calcaneus was held with one hand and the leg was lifted, and the other hand was fixed so that the patient’s knee was not flexed. This test has good validity and reliability (0.94) [24]. In the popliteal angle test, the subject’s knee was extended with the subject was lying in the supine position and the hip joint flexed to 90°. This test has good validity and reliability (0.75) [13].

### 2.5. Data Analysis

Data analysis was performed using SPSS ver. 24.0 (SPSS Inc., Chicago, IL, USA). The data were presented as means and standard deviations. The Shapiro–Wilk normality test was performed when all items were normally distributed. The general characteristics of the participants were presented as descriptive statistics. Independent t-tests were used to compare the differences between the groups. A paired *t*-test was used to compare the differences between the groups. The significance level for the analyses was set at 0.05.

## 3. Results

The experimental results showed that all items were homogeneous in the HR group and the control group (Table 1). There was no significant difference between the groups.

### 3.1. Headache

The HR group and control group showed a significant difference before and after training in the HIT-6 and VAS scores (*p* < 0.001), and there was a significant difference in the scores between the HR group and control group (*p* < 0.05) (Table 2).

### 3.2. Pain Pressure Threshold

The HR group and control group showed a significant difference before and after training in the neck (left and right) PPT and hamstrings (left and right) PPT (*p* < 0.05), and there was a significant difference in the scores between the HR group and control group (*p* < 0.05) (Table 3). 

### 3.3. Range of Motion

Regarding neck flexion ROM, the HR group and control group showed a significant improvement before and after training (*p* < 0.05). There was a significant difference in the scores between the HR group and control group (*p* < 0.05). In the SLRT (left and right), the HR group and control group showed a significant improvement before and after training (*p* < 0.05), and the HR group showed a significant difference in the scores compared to the control group (*p* < 0.001). Regarding the popliteal angle test (left and right), the HR group and control group showed a significant improvement before and after training (*p* < 0.05). The HR group showed a significant difference in the scores compared to the control group (*p* < 0.01) (Table 4). 

## 4. Discussion

In this study, 30 patients with tension headache underwent an HR program and physical therapy for 4 weeks. The purpose of this study was to evaluate the effect of the intervenient on the degree of headache, the PPT of the suboccipital and hamstring, and the ROM of the suboccipital muscles and hamstrings.

### 4.1. Headache

Despite the advances in science over the past decade, treatments for headache are still developing. Cervical dysfunction or active pain trigger points lead to tension headaches involving the neck and shoulder muscles. Tension in the suboccipital, upper trapezius, sternoclavicular, temporal, and neck flexor muscles was associated with tension headache [25]. In their study involving 20 patients with tension headache, Bezov et al. [26] reported that the VAS pain score decreased significantly from 27.6 points to 18.4 points as the tension in the upper trapezius and suboccipital muscles decreased (*p* < 0.05).

In this study, the mean headache effect test score showed a significant decrease from 65.93 points to 53.53, and the VAS score significantly decreased from 6.27 points to 2.73 points after the HR program. This means the program had a positive effect on the tension in the neck muscles due to the relaxation of the posterior fascia during the program. The pain pattern of tension headache was caused by pain from the active pain trigger points in the posterior neck, head, and shoulder muscles. Active pain trigger points appear a lot in tense muscles. 

Oliveira-Campelo et al. [27] reported that whether neck pain and headache through relaxation of the pain-inducing points of the masticatory and cervical muscles, autogenous fascia relaxation, and cervical vertebral correction techniques and as a result, a significant difference (*p* < 0.05) was reported after autologous fascia relaxation. In addition, Schleip [28] showed a significant decrease in the mean PPT of the suboccipital muscle after hamstring stretching in 30 patients with hamstring shortening syndrome. Accordingly, the VAS score for headache significantly decreased from 7.45 points to 3.29 points. Many studies have reported that tension in the neck or shoulder muscles is related to headaches. 

In this study, there was a significant difference between the HR group and the control group in the headache effect test and VAS scores (*p* < 0.05). As the posterior fascia connecting the suboccipital muscles to the hamstrings was relaxed, this may have had a positive effect on the pain due to tension headache, considering that the headache was associated with tension in the neck muscles. 

Jagtap et al. [29] reported that prolonged muscle control had a positive impact on the suboccipital muscles, and consequently, on headache and neck pain in patients with hamstring tension syndrome, and there was a significant difference between the control and experimental groups (*p* < 0.05). It is thought that the posterior fascia-based interventions are effective. In this study, the HR group showed significantly higher improvement in headache scores than the control group (*p* < 0.05). One of the common malalignments of headache is forward head posture (FHP). FHP is an excessive anterior head position in relation to a vertical reference line, as manifested by a reduced cranio-vertebral angle (CVA) [30]. The indicated that the presence of FHP can lead to delayed or inhibited activation of the deep neck flexors in the cervical spine, which is often accompanied by shortening of the opposing suboccipital muscles in parallel [31]. What is associated with the forward head posture in the whole body’s malalignment is the flat back and sway-back posture. What is more, the flat back and sway-back posture cause the trunk, pelvis, and lower extremities to center of gravity move backward from the sagittal plane due to the shorter hamstring muscle. Then, the head is moved forward to compensate for the balance of the body moved backward [15]. This occurs because the superficial back line of the myofascial chain connects from the neck (neck and trunk extensor muscles, sub-occipitalis muscles) to the lower extremity (hamstring and calf muscles), and the soft tissue in the cervical spine links the dura and suboccipital muscle fascia [32]. Therefore, since shortening the hamstring muscle causes the body’s inappropriate posture, I think stretching and relaxation of the hamstring muscle should be accompanied to improve the forward head posture, which is one of the main causes of headaches. Hence, the HR program is a more effective intervention for headache than physical therapy.

### 4.2. Pain Pressure Threshold

Tension headaches cause central sensitization in the sensory tissues of the spine when harmful stimuli are received by the fascia tissue of the peripheral muscles [5]. Harmful stimulation of the fascia tissue can cause ischemia due to inadequate muscle function and activity, which is a factor that causing pains. Factors such as muscle tension, tension in skeletal muscles, and increased tenderness in peripheral muscles are important factors in the formation of pain-inducing trigger points. The pain trigger points cause pain and muscle tension, which affect the ROM of the head and cervical joint [26]. Schleip [28] showed that the average PPT of the suboccipital muscle had significantly decreased from 2.21 kg/cm^2^ to 1.82 kg/cm^2^ after patella stretching in 30 patients with hamstring shortening syndrome. Accordingly, the VAS score for headache had significantly decreased from 7.45 points to 3.29 points.

In this study, after the HR program, there was a significant increase in the mean PPT from 1.47. to 1.83 kg/cm^2^. This is thought to have a positive effect when the PPT is measured as it affects the tension of the suboccipital muscle connected to the posterior fascia when the hamstring was relaxed. Fernández et al. [33] measured the tension in the cervical spine and masticatory muscles using the SLRT. In the experimental group, the mean PPT increased from 2.69 kg/cm^2^ to 4.24 kg/cm^2^, showing a significant difference in the pre- and post-intervention scores (*p* < 0.05). According to Aparicio [13], the cervical muscle suppression technique led to a significant increase in the mean PPT of the hamstrings from 6.78 kg/cm^2^ to 7.34 kg/cm^2^, and significant improvement in the SLRT result; from 59.22° to 65.11°.

In this study, the HR program significantly increased the PPT of the left side hamstrings from 2.84 kg/cm^2^ before the experiment to 3.46 kg/cm^2^ and the PPT of the right-side hamstrings from 2.68 kg/cm^2^ before the experiment to 3.20 kg/cm^2^. This is the HR program, which has a positive effect on the fascia and muscles of the hamstring, and it is thought that this has a positive effect on the degree of tension headache affected by the tension in the suboccipital or trapezius muscles. Moreover, in this study, there was a significant decrease in the PPT of both the left and right muscles suboccipital muscles and hamstring in both the HR and the control groups (*p* < 0.05). These results are considered to HR program was conducted, and the relaxation of the posterior fascia affected the relaxation of the suboccipital muscle, and it was thought that a significant result was obtained in the PPT. In addition, since the HR program was performed on both the left and right sides.

Based on the results, the HR program is effective in relieving the PPT of the suboccipital muscles and hamstrings. The HR group showed a more significant difference in the PPT of the suboccipital muscles and hamstrings than the control group (*p* < 0.05). The patients with FHP exhibit weakness in the extensor (lower parts of semispinalis, splenius, and upper trapezius) and flexor muscles (longus colli, longus capitus, and rectus capitis anterior) of the neck, as well as shortening of the suboccipital and sternocleidomastoid muscles [34]. Such changes lead to increased pressure between the cervical vertebrae and the development of tender points within the neck region [7]. The improved pressure pain threshold is believed to be due to increased muscle length of the hamstring muscles, resulting in improved flat back and sway-back posture, which corrected the forward head posture to a normal position. The patients with FHP exhibit weakness in the extensor (lower part of semispinalis, splenius, and upper trapezius) and flexor muscles (longus colli, longus capitus, and rectus capitis anterior) of the neck, as well as shortening of the suboccipital and sternocleidomastoid muscles. Hence, the HR program can be effective in improving the PPT of the muscles along with physical therapy.

### 4.3. Range of Motion

Exercise programs, including stretching, are very helpful in maintaining muscle flexibility and preventing injury, and ROM exercises can have a positive effect on musculoskeletal health. Functional limitations in the muscles and fascia due to disease, restriction of movement, or inflammation can negatively affect flexibility, muscle strength, endurance, and coordination [35]. In many previous studies, changes in the hamstrings were measured when neck interventions were made, but few previous studies measured the ROM of the neck when hamstring interventions were made. In this study, we found a significant difference in the ROM of the cervical spine flexion before and after the HR program. This is thought to be due to relaxation of the posterior fascia that connects the muscles and fascia when the neck is relaxed and when the hamstrings are relaxed. Jagtap et al. [29] reported a significant improvement of 16° in the SLRT result due to the relaxation of neck tension (*p* < 0.05). It was found that changes in hamstring length and relaxation of the occipital muscles are mutually effective therapeutic interventions. In addition, Pollard and Ward [36] found that the hamstrings were relaxed after applying cervical vertebrae relaxation techniques. Schleip [28] performed proprioceptive neuromuscular facilitation techniques on the muscles on the back of the cervical spine and hamstrings and reported a significant difference (*p* < 0.05) in the SLRT results before and after the intervention. In addition, Fernández et al. [33] measured the changes in SLRT results after applying interventions for tension in the cervical spine and masticatory muscles. In the experimental group, as the PPT decreased, and the SLRT result increased by an average of 14° (*p* < 0.05). In this study, when the HR program was applied, a significant increase was found in the SLRT results from 60.78° before to 72.16° after the experiment. It is thought that a significant difference was found in hip flexion, that is, the straight leg elevation test, which is affected by the muscle tension of the proximal hamstring as stretching the proximal part of the hamstring during the HR program.

Jagtap et al. [29] found a significant difference in the knee angle test and anterior flexion distance test results before and after applying the cervical muscle suppression technique in patients with shortened hamstrings (*p* < 0.05). Cho et al. [30] reported that the self-fascial relaxation technique of the cervical spine led to a significant improvement in the knee angle test and the SLRT results in patients with shortened hamstrings (*p* < 0.05). Aparicio et al. [13] found that the cervical muscle suppression intervention significantly increased the SLRT result from 59.22° to 65.11°. In addition, the knee angle test result increased significantly from 121.97° to 117.83° (*p* < 0.05).

In this study, there was a significant increase in the knee angle test result from 138.69° before to 156.10° after the HR program. This result can be attributed to the stretching of the distal part of the hamstring during the HR program. In this study, there was a significant increase in neck flexion ROM and leg elevation test and knee angle test results in both the HR program group and the control group (*p* < 0.05) after the interventions, mainly because the HR program was performed by subdividing the hamstring into the distal and proximal parts. In the study by Cho [9], a significant difference was found in the SLRT and knee angle test results before and after the interventions.

As the hamstring relaxes, the relaxation of the posterior fascia affects the occipital muscle, and this improves the flexion range of the neck. Thus, while both intervention methods are effective in increasing the ROM of the suboccipital muscles and hamstrings, the HR group showed more significant differences than the control group, suggesting that the HR program is a more effective intervention method for improving the ROM of the suboccipital and hamstrings than physical therapy.

The limitations of this study are as follows: First, the degree of headache was subjectively measured by the individuals. In this study, the limitations of muscle function were measured only by the ROM and PPT and no other detailed measurements were recorded. In addition, the HR program was applied to both the left and right sides, but all patients do not have the same muscle tension or function on both sides, so it is not clear whether the HR program had a better effect on the more tensed side than on the contralateral side. Second, the number of participants in this study are thirty, which is somewhat insufficient as a representative study that can generalize the population around the world. It is difficult to determine the effects of gender differences and age on the results. TTH occurs more often in women than in men and some studies have suggested an increase during hormonal changes such as menses or pregnancy [37]. The effect of menopause on TTH is less clear than the corresponding effect on migraine [38]. A prevalence peak is reached in women between the ages of 35 and 45, with 25–30% of the general female population being affected, in comparison to only 8% of the general male population [39]. However, in this study, it is not known how the difference according to gender and age of TTH affects the outcome. Nevertheless, I think it is meaningful to prove the reduction of headaches and the improvement of ROM in TTH patients through HR training. In the future, we will study the differences according to gender and age of TTH through continuous research. Other events may have occurred during the program, which were not controlled. In future studies, these limitations must be considered.

## 5. Conclusions

We found that the HR program was effective in reducing the degree of headache in patients with tension headache and in improving the PPT and ROM of the suboccipital muscles and hamstrings. Hence, the HR program can be considered effective in alleviating headaches. In the future, HR programs can include improvements in the work environment to achieve improvements in patients with tension headache.

## Figures and Tables

**Table 1:** General characteristics of participants (N = 30).

Characteristics	HR Group (*n* = 15)	Control Group (*n* = 15)	*t*	*p*/x^2^
Sex (male/female)	8/7	7/8	0.354	0.726
Ages (years)	37.93 ± 10.32	38.27 ± 11.10	0.085	0.933
Height (cm)	170.87 ± 7.16	169.87 ± 8.59	0.346	0.732
Weight (kg)	66.13 ± 14.09	64.47 ± 11.99	0.349	0.730

Values are expressed as mean ± standard deviation. HR group, hamstring relaxation group.

**Table 2:** Comparison of headache intensity (N = 30).

Headache	HR Group (*n* = 15)	Control Group (*n* = 15)	*t*	*p*
HIT-6 score				
pretest	65.93 ± 3.95	63.80 ± 3.40	1.583	0.125
posttest	53.53 ± 3.72	56.07 ± 6.26		
pre-post	11.73 ± 4.77	7.73 ± 5.49	2.128	0.042
*t*(*p*)	7.610 (0.000)	5.449 (0.000)		
VAS score				
pretest	6.27 ± 1.38	5.33 ± 1.17	1.988	0.057
posttest	2.73 ± 1.28	3.13 ± 1.45		
pre-post	3.53 ± 1.19	2.20 ± 1.65	2.534	0.017
*t*(*p*)	11.526 (0.000)	5.145 (0.000)		

Values are expressed as mean ± standard deviation. HR group, hamstring relaxation group; HIT-6, headache impact test-6; VAS, visual analogue scale.

**Table 3:** Comparison of pain pressure threshold (N = 30).

PPT	HR Group (*n* = 15)	Control Group (*n* = 15)	*t*	*p*
Lt neck PPT (kg/cm^2^)				
pretest	1.47 ± 0.43	1.55 ± 0.31	−0.545	0.590
posttest	1.83 ± 0.45	1.70 ± 0.31		
pre-post	0.36 ± 0.29	0.15 ± 0.23	2.132	0.042
*t*(*p*)	−4.719 (0.000)	−2.558 (0.023)		
Rt neck PPT (kg/cm^2^)				
pretest	1.37 ± 0.46	1.50 ± 0.34	−0.883	0.385
posttest	1.87 ± 0.47	1.58 ± 0.26		
pre-post	0.50 ± 0.27	0.07 ± 0.12	5.501	0.000
*t*(*p*)	−7.199 (0.000)	−2.433 (0.029)		
Lt hamstring PPT (kg/cm^2^)				
pretest	2.84 ± 0.72	3.12 ± 0.75	−1.026	0.314
posttest	3.46 ± 0.93	3.25 ± 0.75		
pre-post	0.61 ± 0.56	0.15 ± 0.21	2.972	0.006
*t*(*p*)	−4.270 (0.001)	−2.210 (0.044)		
Rt hamstring PPT (kg/cm^2^)				
pretest	2.68 ± 0.75	2.94 ± 0.87	−0.860	0.397
posttest	3.20 ± 0.86	3.04 ± 0.88		
pre-post	0.51 ± 0.40	0.10 ± 0.12	3.765	0.001
*t*(*p*)	−4.945 (0.000)	−3.390 (0.004)		

Values are expressed as mean ± standard deviation. HR group, hamstring relaxation group; PPT, pressure pain threshold; Rt, right side; Lt, left side.

**Table 4:** Comparison of the range of motion (N = 30).

ROM	HR Group (*n* = 15)	Control Group (*n* = 15)	*t*	*p*
Neck flexion ROM (°)				
pretest	49.16 ± 10.77	47.70 ± 11.23	0.363	0.719
posttest	53.94 ± 10.02	50.08 ± 11.33		
pre-post	4.78 ± 2.61	2.38 ± 3.53	2.120	0.043
*t*(*p*)	−7.092 (0.000)	−2.607 (0.021)		
Lt SLRT (°)				
pretest	60.78 ± 11.17	58.66 ± 9.88	0.549	0.587
posttest	72.16 ± 11.84	60.64 ± 10.01		
pre-post	11.38 ± 5.59	1.97 ± 3.36	5.576	0.000
*t*(*p*)	−7.873 (0.000)	−2.269 (0.004)		
Rt SLRT (°)				
pretest	60.26 ± 11.29	59.57 ± 10.89	0.169	0.867
posttest	74.29 ± 10.88	61.98 ± 9.20		
pre-post	14.03 ± 8.14	2.41 ± 4.23	4.904	0.000
*t*(*p*)	−6.674 (0.000)	−2.208 (0.044)		
Lt PAT (°)				
pretest	138.69 ± 19.24	150.08 ± 14.40	−1.835	0.077
posttest	156.10 ± 14.63	155.98 ± 11.73		
pre-post	17.41 ± 12.92	5.90 ± 9.57	2.772	0.010
*t*(*p*)	−5.218 (0.000)	−2.385 (0.032)		
Rt PAT (°)				
pretest	141.45 ± 18.42	152.07 ± 13.99	−1.778	0.086
posttest	155.28 ± 14.16	154.60 ± 13.57		
pre-post	13.82 ± 12.26	2.52 ± 4.52	3.347	0.002
*t*(*p*)	−4.365 (0.001)	−2.164 (0.048)		

Values are expressed as mean ± standard deviation. HR group, hamstring relaxation group; ROM, range of motion; SLRT, straight leg raise test; PAT, popliteal angle test; Rt, right side; Lt, left side.

## Data Availability

Not applicable.
